# Case Report: Giant pheochromocytoma complicated by takotsubo syndrome: a case of emergency robot-assisted left adrenalectomy and multidisciplinary management

**DOI:** 10.3389/fonc.2025.1603477

**Published:** 2025-06-16

**Authors:** Jia Miao, Xuanhan Hu, Ting Wang, Feng Liu, Dahong Zhang, Haibin Wei

**Affiliations:** ^1^ The Second School of Clinical Medicine, Zhejiang Chinese Medical University, Hangzhou, Zhejiang, China; ^2^ Urology & Nephrology Center, Department of Urology, Zhejiang Provincial People’s Hospital, Affiliated People's Hospital, Hangzhou Medical College, Hangzhou, Zhejiang, China

**Keywords:** pheochromocytoma, robotic surgery, Takotsubo syndrome, emergency surgery, multidisciplinary management

## Abstract

Pheochromocytomas are rare neuroendocrine tumors that can cause life-threatening cardiovascular complications due to excessive catecholamine secretion. One such severe manifestation is Takotsubo syndrome (TS), a catecholamine-induced cardiomyopathy that exacerbates hemodynamic instability. The coexistence of a giant pheochromocytoma and TS is extremely rare and presents formidable diagnostic and therapeutic challenges. This case highlights the successful emergency management of this complex condition, demonstrating the feasibility of robotic adrenalectomy in hemodynamically unstable patients. A 45-year-old male presented with dizziness, headache, chest tightness, and palpitations. He was found to have severe hypertension (220/130 mmHg), elevated cardiac biomarkers (BNP 114 pg/mL, cTnI 0.046 ng/mL), and a large left adrenal mass (119 mm × 139 mm × 130 mm). During hospitalization, he experienced alternating hypertensive crises (peak 261/151 mmHg) and profound hypotension (54/32 mmHg), with echocardiography revealing apical ballooning and reduced ejection fraction (45%), consistent with TS. Biochemical analysis showed markedly elevated plasma catecholamine. Given the severe hemodynamic instability despite intensive medical management, an emergency robot-assisted left adrenalectomy was operated. Intraoperatively, significant blood pressure fluctuations occurred (peak 230/100 mmHg), requiring additional vasoactive agent. Pathologic examination confirmed a 14 cm pheochromocytoma. Postoperatively, cardiac function normalized within three months, and catecholamine levels returned to baseline. This case highlights the critical association between pheochromocytoma and TS, underscoring the potential for severe hemodynamic instability in such patients. The successful use of urgent robotic adrenalectomy demonstrates its feasibility and advantages in managing giant pheochromocytoma, even in high-risk patients. This report contributes to the growing evidence supporting minimally invasive techniques in endocrine surgery and emphasizes the necessity of multidisciplinary collaboration in optimizing outcomes for complex pheochromocytoma cases.

## Introduction

Pheochromocytoma is a rare neuroendocrine tumor originating from the adrenal medulla, characterized by its ability to synthesize and store catecholamines, including epinephrine and norepinephrine (1). The episodic release of these hormones triggers the classic triad of headache, sweating, and palpitations. In certain patients, catecholamine-induced cardiomyopathy may mimic Takotsubo cardiomyopathy, leading to severe hemodynamic instability. Given the potential severity of this condition, accurate diagnosis and timely intervention are critical for effective management.

## Case presentation

A 45-year-old male presented to the emergency department with a one-day history of dizziness, headache, vomiting, chest tightness, palpitations, tinnitus, and transient visual disturbances. He had previously sought medical attention at another hospital, where his blood pressure was recorded as high as 220/130 mmHg. The patient had no known history of cardiovascular disease, diabetes, or other chronic illnesses. Prior to this episode, he had not undergone routine blood pressure monitoring and had never sought medical attention for symptoms such as headache, palpitations, or dizziness. He had not received any antihypertensive therapy before. This presentation marked his first documented hypertensive crisis. Laboratory tests revealed a BNP level of 114 pg/mL and a cardiac troponin I (cTnI) level of 0.046 ng/mL. A computed tomography (CT) scan identified a mass in the left adrenal region.

Upon admission to our institution, his vital signs were as follows: temperature, 36.0°C; blood pressure, 141/93 mmHg; oxygen saturation, 97%; and a heart rate, 86 beats per minute. Laboratory tests demonstrated elevated levels of Pro-BNP (1020 pg/mL, normal <133 pg/mL), BNP (537.5 pg/mL; normal <87 pg/mL), cTnI (4.239 μg/L; normal <0.050 μg/L), and creatine kinase (CK) (392 U/L; normal range: 55–170 U/L). Routine blood tests, including complete blood count, renal and liver function tests, serum electrolytes, and coagulation profile, were within normal ranges and thus were not included in detail. Echocardiography revealed blunting of the left ventricular apex, diffuse hypokinesis of the mid-to-apical segments, and reduced left ventricular systolic function with an ejection fraction of 45%. The patient was managed with antihypertensive therapy, gastroprotection, acid suppression, and antiemetics.

**Figure 1:**
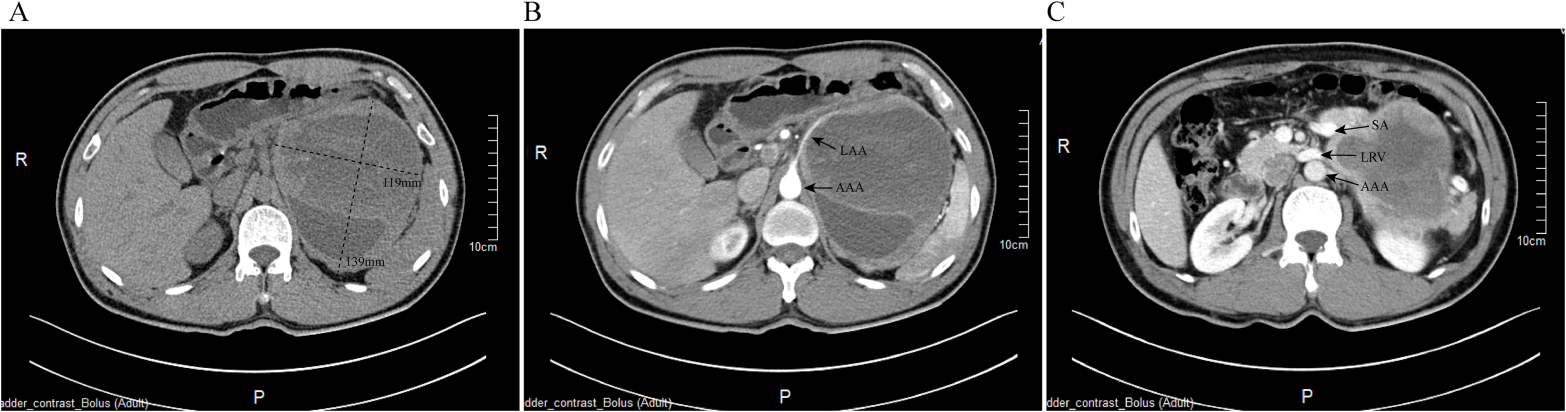
Preoperative contrast-enhanced CT imaging of the left adrenal pheochromocytoma. **(A)** Non-contrast axial CT scan showing the adrenal mass measuring approximately 119 mm × 130 mm; **(B)** Arterial phase imaging demonstrating the tumor’s vascular supply, with the left adrenal artery (LAA) originating from the abdominal aorta (AAA); **(C)** Venous phase imaging highlighting tumor involvement with surrounding vasculature, including the splenic artery (SA), left renal vein (LRV), and AAA.

Plasma free catecholamine levels and urinary catecholamine analysis were markedly elevated (**Table 1**). Coronary angiography ruled out ischemic heart disease. Abdominal contrast-enhanced CT revealed a large left adrenal mass measuring 119 mm × 139 mm × 130 mm (**Figure 1**). Electrocardiogram (ECG) demonstrated T-wave abnormalities, and Holter monitoring detected 360 atrioventricular junctional escape beats, T-wave changes, and QT prolongation, consistent with catecholamine-induced cardiomyopathy resembling Takotsubo syndrome (TS).

**Table 1:** Preoperative and postoperative biochemical profiles of catecholamine metabolites.

Biomarker	Preoperative Level	Postoperative Level	Reference Range
**FMN (pg/mL)**	>500.0	30.2	0-62
**FNMN (pg/mL)**	>1000.0	374.4	0-145
**FMN + FNMN (pg/mL)**	>1500.0	404.6	0-207
**Free Epinephrine (μg/day)**	103.9	4.1	0-20
**Free Norepinephrine (μg/day)**	493.7	41.0	0-90
**Dopamine (μg/day)**	290.1	278.2	0-600
**Metanephrine (μg/day)**	>1960.0	59.6	62-207
**Normetanephrine (μg/day)**	>3920.0	204.7	125-510
**VMA (mg/day)**	>61.3	5.9	0-7.5

FMN, Free Metanephrine; FNMN, Free Normetanephrine; VMA, Vanillylmandelic Acid.

Initially, elective surgical intervention was planned with preoperative optimization focusing on blood pressure control, volume expansion, and heart rate stabilization. However, on the ninth day of hospitalization, the patient experienced severe hemodynamic instability characterized by alternating hypertensive and hypotensive crises, with blood pressure fluctuating dramatically from 261/151 mmHg to 54/32 mmHg within a span of just 20 minutes. These rapid oscillations, coupled with worsening clinical symptoms and persistently elevated cTnI (1.826 μg/L) and BNP (909.8 pg/mL) levels, necessitated immediate transfer to the intensive care unit (ICU) for intensive monitoring and urgent surgical intervention. Given his deteriorating condition, he was transferred to the ICU for intensive monitoring and preparation for emergency surgery.

**Figure 2:**
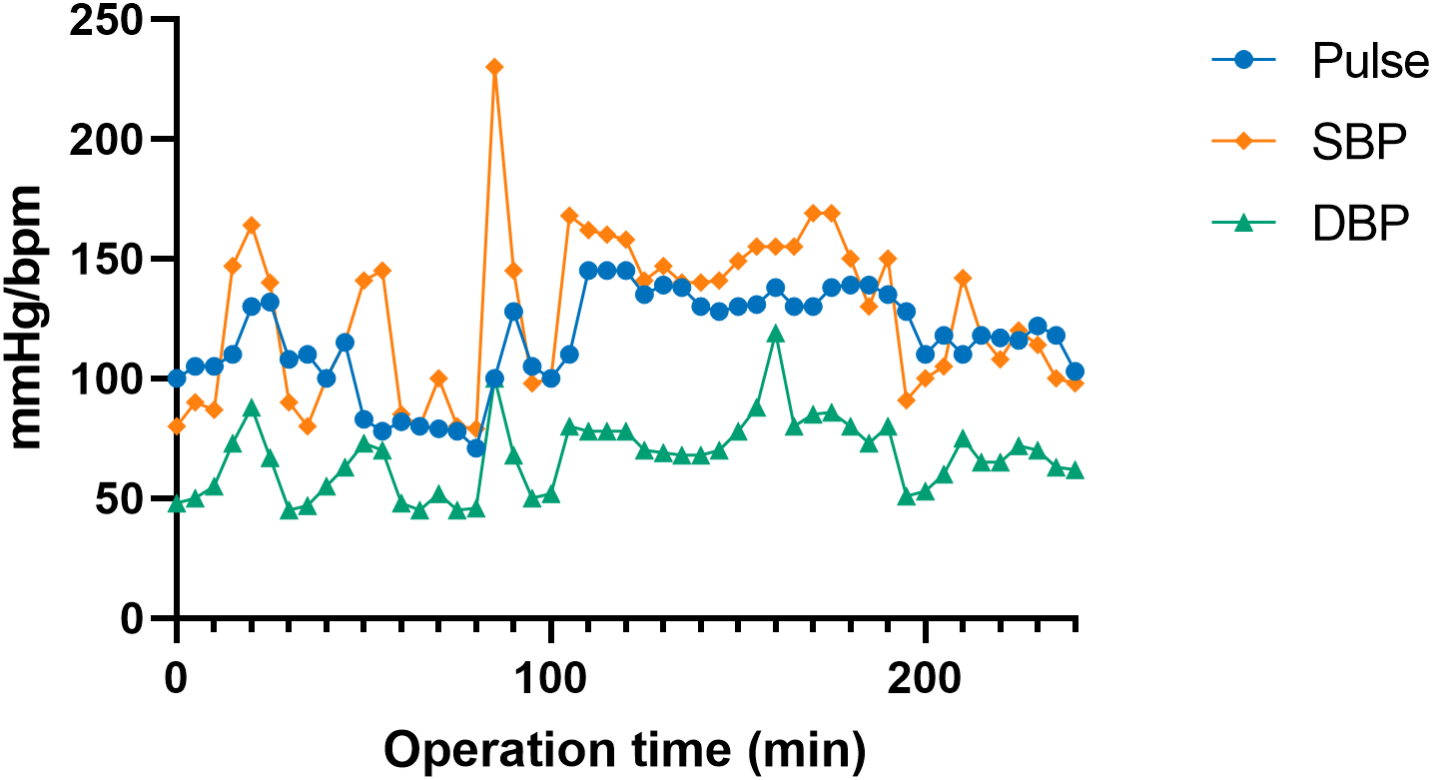
Intraoperative hemodynamic fluctuations during robot-assisted adrenalectomy. The graph illustrates the fluctuations in heart rate (Pulse), systolic blood pressure (SBP), and diastolic blood pressure (DBP) throughout the surgical procedure.

On the tenth day of hospitalization, an emergency robot-assisted laparoscopic left adrenalectomy was performed. Intraoperatively, tumor invasion into the psoas major muscle was observed. Blood pressure fluctuations were significant, with a peak of 230/100 mmHg during tumor manipulation (**Figure 2**). Intraoperative management included total fluid replacement of 6500 mL, transfusion of 370 mL of plasma, and an estimated blood loss of 300 mL. Phentolamine was administered for blood pressure control. The patient was extubated on the first postoperative day, and the drainage tube was removed on postoperative day nine. Follow-up laboratory tests demonstrated improvement, with BNP decreasing to 290.4 pg/mL and cTnI to 0.041 μg/L. Urinary catecholamine levels also normalized (**Table 1**).

**Figure 3:**
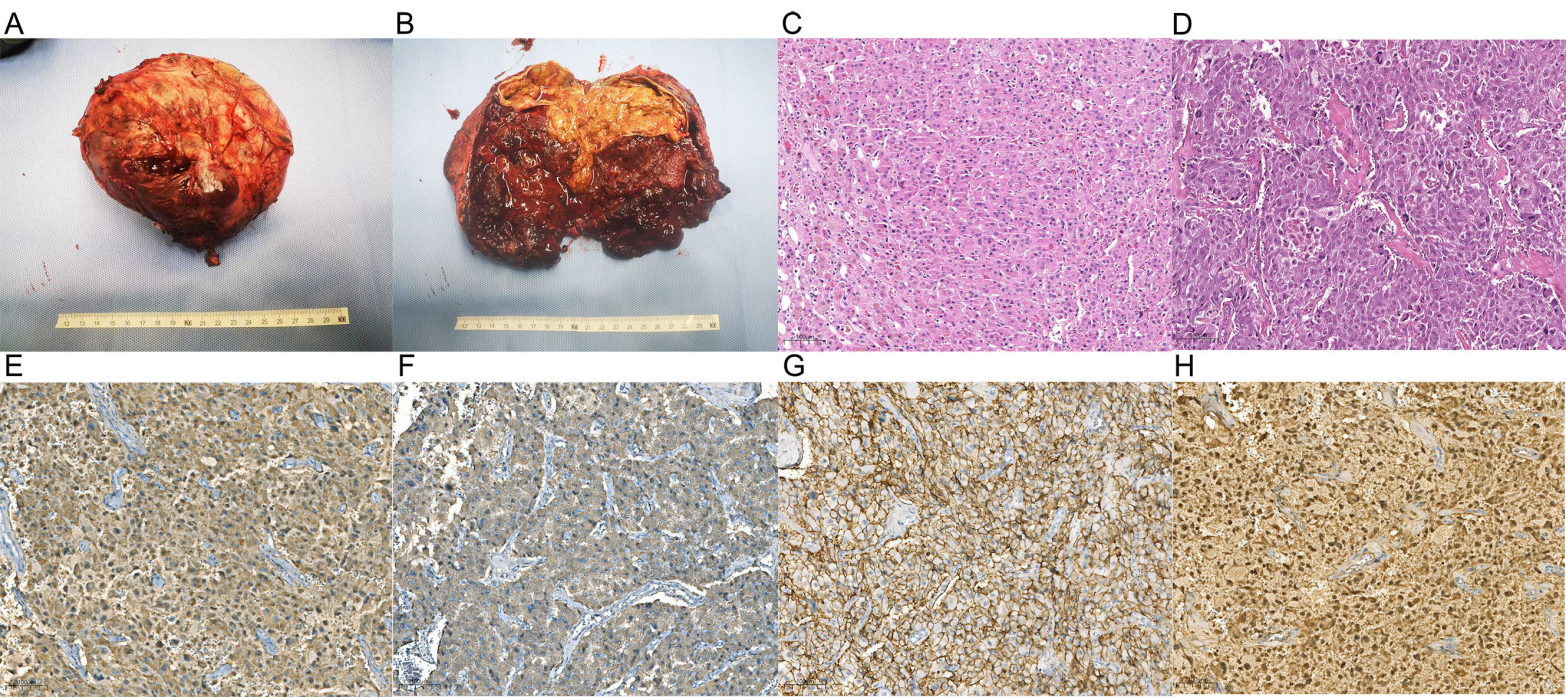
Macroscopic and microscopic characteristics of the adrenal tumor. **(A)** Gross pathological specimen of the tumor; **(B)** Cross-sectional view of the tumor after dissection; **(C)** normal adrenal tissue; **(D)** tumor cells demonstrating Zellballen growth pattern and nuclear atypia; **(E)** synaptophysin positive; **(F)** chromogranin A positive; **(G)** CD56 positive; **(H)** S-100 positive; Synaptophysin, chromogranin A, and CD56 are neuroendocrine markers, and S-100 is a marker of neural tissue.

Pathologic examination confirmed a left adrenal pheochromocytoma measuring 14 cm × 12 cm × 10 cm, with focal invasion into surrounding fibroadipose tissue (**Figure 3**). Immunohistochemical staining showed tumor cells positive for SYN, CgA, CD56, S-100 and SDHB, with no SDHB loss. Ki67 was low (<1%).

At 1, 3, and 10 months postoperatively, the patient remained normotensive, and his cardiac biomarkers, ECG, and echocardiography findings had normalized by the third month.

## Discussion

This case highlights a rare presentation of catecholamine-induced TS associated with a massive pheochromocytoma. Although the patient did not experience typical chest pain or electrocardiographic changes indicative of ST-elevation myocardial infarction, the presence of elevated cardiac biomarkers and T-wave abnormalities initially raised concerns for acute coronary syndrome. However, subsequent coronary angiography demonstrated unobstructed coronary arteries, and transthoracic echocardiography revealed characteristic apical ballooning and hypokinesis extending from the mid to apical segments—hallmarks of Takotsubo syndrome. Taken together with markedly elevated catecholamine levels, these findings supported the diagnosis of catecholamine-induced Takotsubo cardiomyopathy. This patient’s course was further complicated by severe hemodynamic instability, which necessitated urgent intervention.

Pheochromocytoma is a neuroendocrine tumor originating from chromaffin cells in the adrenal medulla. It frequently leads to excessive secretion of epinephrine and norepinephrine, triggering an adrenergic crisis characterized by paroxysmal hypertension, the classic triad of headache, sweating, and palpitations, as well as tachyarrhythmias and myocardial dysfunction (2–5). In this case, the extreme blood pressure fluctuations, ranging from hypertensive crises to profound hypotension, underscore the severity of autonomic instability associated with large pheochromocytomas.

Excessive catecholamine release can induce acute, reversible left ventricular wall motion abnormalities (LVWMA), a hallmark feature of TS, also known as neurogenic myocardial stunning or broken heart syndrome. TS is characterized by regional and circumferential LVWMA, typically presenting as balloon-like dilation of the left ventricle during systole (6). Clinically, TS often mimics acute coronary syndrome (ACS), with overlapping features on ECG and elevated “myocardial infarction biomarkers” (7). Common ECG patterns in TS include ST-segment elevation resembling ST-elevation myocardial infarction (STEMI), ST-segment depression, T-wave inversion, and QT interval prolongation (8, 9). Troponin levels are elevated in 71%-95% of TS cases; however, they are generally lower than those observed in ACS and do not correspond proportionally to the severity of left ventricular dysfunction. Echocardiography often reveals classic features such as apical ballooning, hypokinesis or akinesis, and occasionally mid-ventricular hypokinesis (8–10). Notably, left ventricular dysfunction in TS often extends beyond a single coronary artery territory but can fully reverse within hours to weeks following effective intervention (6, 11). In our case, the patient’s cTnI levels normalized by the ninth postoperative day, and both echocardiographic and ECG findings had returned to normal by the three-month follow-up.

Surgical resection remains the optimal treatment for pheochromocytoma, with available approaches including open surgery, laparoscopic surgery, and robot-assisted laparoscopic surgery. Currently, laparoscopic surgery is considered the gold standard for benign and secretory adrenal lesions (12, 13). However, tumors larger than 6 cm or those with invasive features have traditionally been managed via open adrenalectomy due to concerns regarding tumor rupture and catecholamine storm (14). Robotic-assisted adrenalectomy offers enhanced precision, improved dexterity, and superior visualization, making it a viable alternative for large and complex adrenal tumors (15). Minimally invasive adrenal surgery can be performed via either a transperitoneal or retroperitoneal approach. Current evidence suggests that the laparoscopic approach is not associated with increased hemodynamic instability (16, 17). Recently, the Society of American Gastrointestinal and Endoscopic Surgeons (SAGES) recommended the retroperitoneal approach for patients with a history of abdominal surgery or those undergoing bilateral adrenalectomy, while the transperitoneal approach is preferred for obese patients or those with tumors larger than 6 cm (18).

In the perioperative management of pheochromocytoma, hemodynamic stability is a primary concern for both surgeons and anesthesiologists. Preoperative alpha-blockade and volume expansion are essential to mitigate intraoperative catecholamine surges and postoperative hypotension (19–21). Additionally, approximately 80% of patients require vasoactive therapy during surgery, including the use of nicardipine, esmolol, or norepinephrine (19). During pheochromocytoma surgery, manipulation of the adrenal gland and tumor can trigger life-threatening acute intraoperative catecholamine release. In this case, significant blood pressure spikes were observed upon tumor contact, prompting an immediate pause in surgery and cessation of epinephrine and norepinephrine administration. Nicardipine was administered for blood pressure control, and phentolamine (500 μg) was added for vasodilation. Early ligation of the central adrenal vein and surrounding tumor vessels, with the anesthesiologist’s consent, is crucial to minimize catecholamine release and avoid unnecessary tumor manipulation. Furthermore, the rapid decline in catecholamine levels following venous ligation or tumor resection may lead to severe hypotension and shock, necessitating aggressive fluid resuscitation and blood pressure support. Anesthesia recovery should also be conducted with appropriate management and close monitoring (22).

Although TS represents a severe cardiac complication, perioperative outcomes in these patients are generally favorable once hemodynamic stabilization is achieved (23). While the standard approach typically involves delaying surgical resection until hemodynamic stability is achieved, our patient’s clinical condition deteriorated rapidly, with dramatic blood pressure fluctuations, worsening cardiac biomarkers, and evidence of end-organ hypoperfusion. In light of the escalating severity and the ongoing risk posed by the large catecholamine-secreting tumor, the multidisciplinary team determined that emergency adrenalectomy was necessary. It was concluded that the dangers of postponing surgery outweighed the risks associated with proceeding urgently, especially when performed by an experienced surgical and anesthetic team. Therefore, emergency surgery is imperative for patients with refractory, life-threatening acute hemodynamic instability, and their perioperative management requires meticulous attention. Given the time-consuming preparation and calibration of robotic surgical systems, as well as the need for specialized operators, robotic surgery is often not the first choice for emergency procedures. In this case, considering the patient’s life-threatening acute hemodynamic instability and the large size of the tumor, robotic surgery was deemed the most beneficial approach, balancing patient safety and minimally invasive principles. With the coordination of a multidisciplinary team, our robotic surgery team responded promptly and collaborated with the surgical team to successfully perform an emergency adrenal tumor resection. Although the roles of vasoactive therapy and circulatory support remain incompletely defined, extracorporeal membrane oxygenation (ECMO) and adrenalectomy are often the treatments of choice in the most severe cases (24, 25).

## Conclusion

This case highlights the importance of considering pheochromocytoma in patients presenting with TS. While thorough preoperative optimization is ideal, emergency surgery may be required in cases of life-threatening hemodynamic instability. This report demonstrates that emergency robotic-assisted adrenalectomy is a viable and effective treatment option when performed by an experienced multidisciplinary team.

## Data Availability

The original contributions presented in the study are included in the article/supplementary material. Further inquiries can be directed to the corresponding author.
